# Onset and duration of transitions into Greenland Interstadials 15.2 and 14 in northern China constrained by an annually laminated stalagmite

**DOI:** 10.1038/srep20844

**Published:** 2016-02-10

**Authors:** Wuhui Duan, Hai Cheng, Ming Tan, R. Lawrence Edwards

**Affiliations:** 1 Key laboratory of Cenozoic Geology and Environment, Institute of Geology and Geophysics, Chinese Academy of Sciences, Beijing, 100029, China; 2 Institute of Global Environmental Change, Xi’an Jiaotong University, Xi’an, 710049, China; 3 Department of Earth Sciences, University of Minnesota, Minneapolis, Minnesota, 55455, USA

## Abstract

The onset and duration of abrupt transitions into Dansgaard-Oeschger (DO) events can be studied in detail in Greenland ice cores given the excellent relative uncertainty of its lamina-counting chronology. For other geological archives, however, the shorter intervals are not determined accurately due to lack of clear annual lamina. Here, we present an oxygen isotope record of a stalagmite with well-developed annual lamina from Xinglong Cave, northern China, covering DO 15 and 14. Except for the absence of Greenland Interstadial (GIS) 15.1, the pattern of this record strongly resembles that of Greenland ice cores on millennial scales as well as the detailed centennial-scale cooling excursions within GIS 14. Additionally, the transitions into GIS 15.2 and 14, constrained by lamina counting, lasted 74 and 27 yr, respectively, both of which are in excellent agreement with that of the NGRIP record on the GICC05 timescales (100 ± 6 and 20 ± 1 yr, respectively). The close coupling of abrupt climatic oscillations on millennial to decadal scales between Greenland and northern China implies a rapid atmospheric teleconnection between the North Atlantic and the East Asian Summer Monsoon regions, probably via the westerlies.

The climate over the last glacial period was characterized by a series of abrupt, millennial-scale climate oscillations known as Dansgaard-Oeschger (DO) events, marked by a rapid warming followed by a slow cooling, referred to as Greenland Interstadial (GIS) and Greenland Stadial (GS)^1^,^2^. These rapid climate swings in polar climate associated with transitions between extreme conditions provide an ideal opportunity to explore the forcing mechanisms in the climate system.

First recognized in Greenland ice cores^1^,^2^, DO cycles have since also been identified in a large number of marine and terrestrial records in both hemispheres^3^,^4^,^5^, but they are particularly well expressed in stalagmite records^6^,^7^,^8^,^9^,^10^,^11^. However, as the dating precision and temporal resolution are different between various geological archives, the precise onset and duration of GS-GIS transitions remain controversial. For example, the timing of the onset of GIS 15.2 and 14 in the Hulu^6^, Kleegruben^9^ and Hölloch^11^ stalagmite records lead the analogous events in the NGRIP record, suggesting that the central value of the Greenland Ice Core Chronology 2005 (GICC05)^12^,^13^ during portions of Marine isotope stage 3 (MIS 3) might be slightly too young. Furthermore, the abrupt transitions from GS to GIS conditions, which took place within a few decades, can be precisely determined using Greenland ice-core records due to their excellent relative uncertainty based on the annual lamina-counting chronology^12^,^13^,^14^,^15^,^16^. However, in other non-laminated records, these short intervals can only be estimated by calculating the difference of absolute chronology between the start and the end of the transition which has a much larger relative uncertainty than the ice core records. This limits the ability to assess the teleconnection in the climate system between Greenland and other regions in detail. However, if a stalagmite has clear and continuous visual annual lamina, along with precise dating control, it allows pinpointing the timing and duration of rapid climate changes by a combination of ^230^Th ages and lamina-counting chronology^17^,^18^,^19^. Unfortunately, these sorts of stalagmites are extremely scarce in glacial periods.

In this study, we present a new high precisely dated stalagmite δ^18^O record from Xinglong Cave (Supplementary Fig. S1), which characterizes DO 15 and 14 in detail, for the first time in northern China. The records from southern Chinese caves exhibit some discrepancies during this period. For example, the transition into GIS 14 is gradual in the Hulu record^6^, but sharp in the Wulu record^10^. Meanwhile, the GIS 15 in the Wulu record^10^ shows a ‘double-spike’, while only one peak is apparent in the Hulu record^6^. In addition, this stalagmite (XL-1) is characterized with high uranium and low detrital thorium content (Supplementary Table. S1) and with clear annual lamina (Supplementary Fig. S2). These features allow not only to verify the precise timing of GIS 15.2 and 14, but also to precisely determine the duration of these shorter climate transitions through lamina counting.

## Results

Xinglong Cave (40°29′ N, 117°29′ E, 710 m a. s. l.) was developed in less than 200-m-thick carbonate of the Wumishan formation, Jixian system (Middle Proterozoic), which mainly consists of banded dolomite with some nodular chert. The cave is primarily composed of one large breakdown chamber, about 42 m high. Located within the temperate monsoon climate zone, the cave area typically has cold-dry winters and hot-wet summers. Mean annual temperature and precipitation are 9.1 °C and 512 mm, respectively (AD 1971–2000).

**Figure 1:**
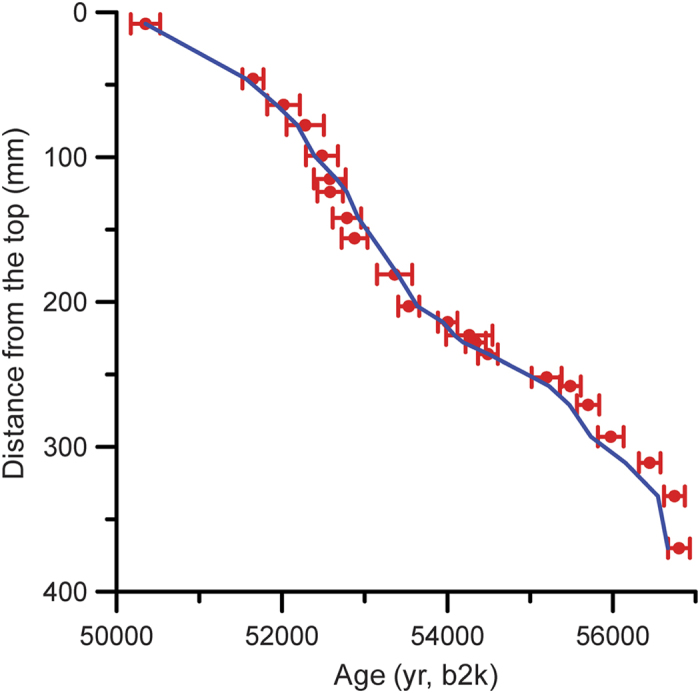
Comparison between ^230^Th age and lamina-counting chronology. The red dots and bars denote the ^230^Th age and 2σ uncertainties, respectively. The blue line denotes the lamina-counting age, the youngest date of which is from that of ^230^Th age.

**Figure 2:**
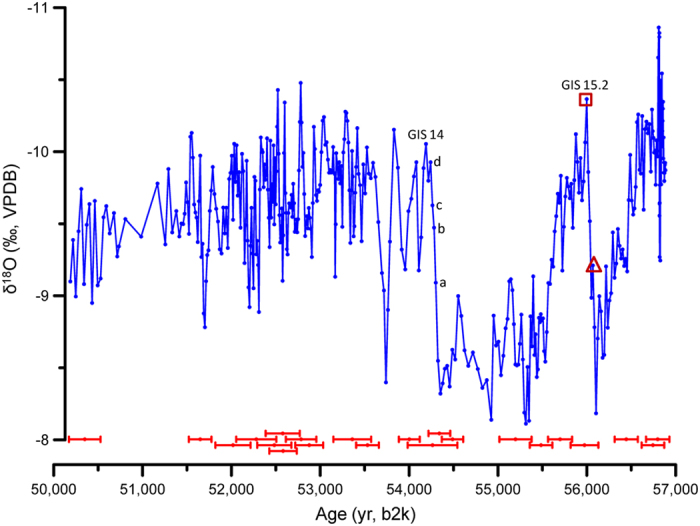
δ^18^O profile of sample XL-1 based on the age model using the StalAge algorithm. ^230^Th dating errors (2σ) are indicated at the bottom. The letters a to d indicate the positions of subsample for δ^18^O analyses during the transition into GIS 14. The triangle and square mark the start and end of transition into GIS 15.2, respectively.

Stalagmite XL-1, 375-mm long, was found naturally broken on the upper part of a collapse slope in the cave, approximately 35 m above the ground surface, where the environment is stable, with a relative humidity close 100% throughout most of the year. 22 high-precision ^230^Th dates were obtained using multi-collector inductively coupled plasma mass spectrometry with typical age uncertainties of less than 0.5% (Supplementary Table S1), showing that stalagmite XL-1 grew continuously from 50.3 to 56.7 ka b2k. One thin section of the whole stalagmite was made to study and count the lamina (Supplementary Fig. S2a). The clear lamina (Supplementary Fig. S2b, c, d) and high-precision ^230^Th dates (Supplementary Fig. S3, Supplementary Table S1) of XL-1 allow a relative chronology to be tied to the ^230^Th dates. Total of 6315 lamina was counted, close to the ^230^Th date (Total 6448 ± 310 yr). Meanwhile, the lamina-counting chronology is in close agreement with each ^230^Th date within errors (Fig. 1), demonstrating that the lamina in XL-1 is annual. 395 sub-samples for δ^18^O analyses were drilled along the growth axis from the thin section using a 0.3 mm dental burr at a sampling interval of 0.5 to 1 mm, with an average temporal resolution of 17 yr (Supplementary Table S2). The δ^18^O values vary between −10.86% and −8.11% (Fig. 2, Supplementary Table S2). A ‘Hendy test’^20^ was performed on four growth lamina with 28 subsamples. Along each single lamina, δ^18^O values are essentially the same, with no statistically significant correlation between δ^18^O and δ^13^C, suggesting that the stalagmite most likely grew close to isotopic equilibrium condition. Hence, the large range in the isotopic values largely reflects changes in the isotopic composition of the local precipitation. The shifts in Chinese stalagmite δ^18^O, in turn, are generally related to changes of East Asian Summer Monsoon (EASM) intensity, with lower δ^18^O values reflecting stronger EASM and vice versa^6^,^8^,^21^,^22^.

**Figure 3:**
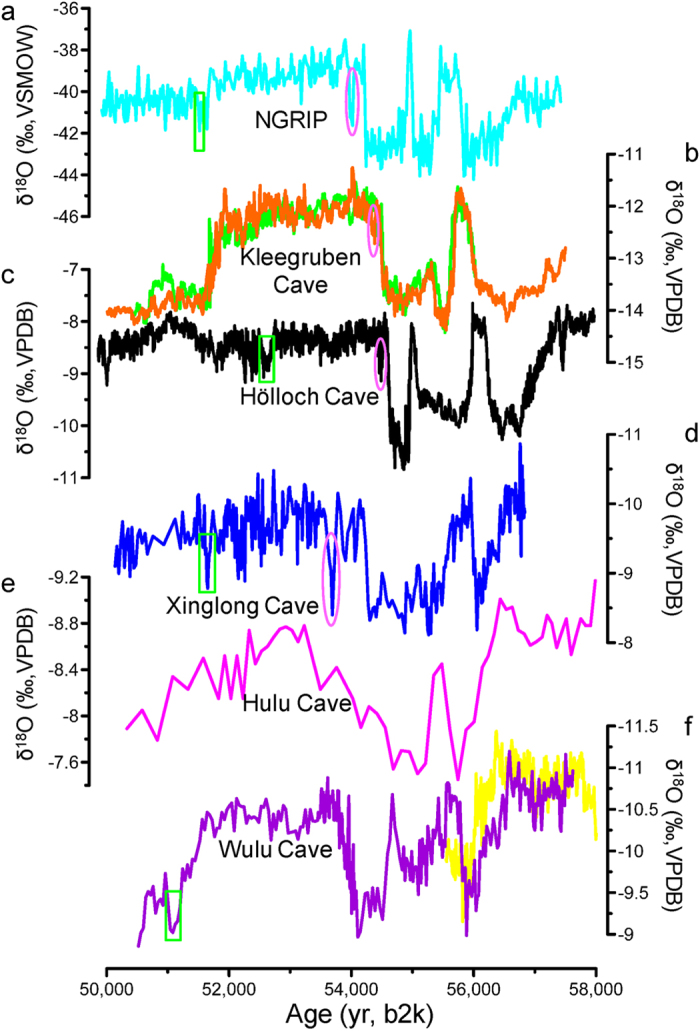
Comparison between different records from Greenland ice core and stalagmites in China and Europe between 50 and 58 ka b2k. (**a)** NGRIP δ^18^O record on the GICC05 timescale (refs 12 and 13). (**b)** Kleegruben Cave stalagmites (green, SPA 126, red, SPA 49, ref. 9). (**c)** Hölloch Cave stalagmites (ref. 11). (**d)** Xinglong Cave stalagmite (this study, based on the StalAge age model). (**e)** Hulu Cave stalagmite (ref. 6). (**f)** Wulu Cave stalagmites (purple, WU 26, yellow, WU 23, ref. 10). The pink ellipse and green rectangle denote centennial-scale climate oscillations within GIS 14 in each record, respectively.

The age model was established using the StalAge algorithm^23^ (Supplementary Fig. S3). The overall pattern of δ^18^O shifts in the XL-1 profile covering DO 15 and 14 is similar to the Hulu and Wulu cave records in southern China^6^,^10^, all of which strongly resemble the Greenland GS-GIS cycles and precursor events, with ^18^O-depleted, stronger-EASM events in Chinese stalagmites^6^,^10^ corresponding to ^18^O-enriched, higher-temperature events in Greenland ice cores^12^,^13^ (Fig. 3). This suggests a tight coupling between the climate in Greenland and China during MIS 3 on millennial timescales. Nevertheless, there are significant differences between the records. For instance, GIS 15 in our record does not show a double spike pattern (GIS 15.1 appears to be absent, possibly because there is a small hiatus which cannot be detected by lamina counting and ^230^Th dating) observed in Greenland ice-core records^12^,^13^,^14^,^16^, European stalagmite records^9^,^11^ and even the Wulu cave record in southern China^10^. The aridity event at the end of GIS 15.2 in our record appears of similar magnitude as the corresponding cold event in the Greenland ice cores^12^,^13^,^14^,^16^, but, is only very weakly expressed in the Wulu record^10^. In general, compared to the cave records in southern China, the overall pattern of our record is more similar to the Greenland ice cores. Additionally, the amplitude of shifts within GIS 14 in our record is larger than in other stalagmite records from southern China (Fig. 3). One possible reason might be that Xinglong Cave is located at the margin of the EASM and may be more sensitive to small changes in the position of the monsoon.

According to the criteria outlined in previous ice core^16^ and stalagmite^11^ studies, the onset of GIS events was defined as the first data point of the steep rise that clearly deviates from the base level of the preceding stadial. The end of each abrupt climate transition was defined as the last data point of the steep rise. The interval between the first and last data point is the duration of the GS-GIS transition (Fig. 2).

The onsets of GIS 15.2 and 14 are well preserved in the stalagmite XL-1 record, with δ^18^O values decreasing sharply to a minimum at the beginning of the GIS, followed by a gradual increase throughout the GIS-GS until the next sharp decrease. The onsets of GIS 15.2 and 14 in our record occurred at 56069 ± 150 and 54300 ± 157 yr b2k, respectively, both in excellent agreement with GICC05^12^,^13^, with less than 270 yr difference (269 yr for GIS 15.2 and 80 yr for GIS 14). Hence our results support the accuracy of the GICC05 timescales in this time range.

Furthermore, as the stalagmite in this study has very clear visual annual lamina, we established an age model of annual resolution in order to determine the duration of the abrupt climate transitions to close to annual precision. This allows a detailed comparison of abrupt transitions into GIS 15.2 and 14 with the NGRIP record.

**Figure 4:**
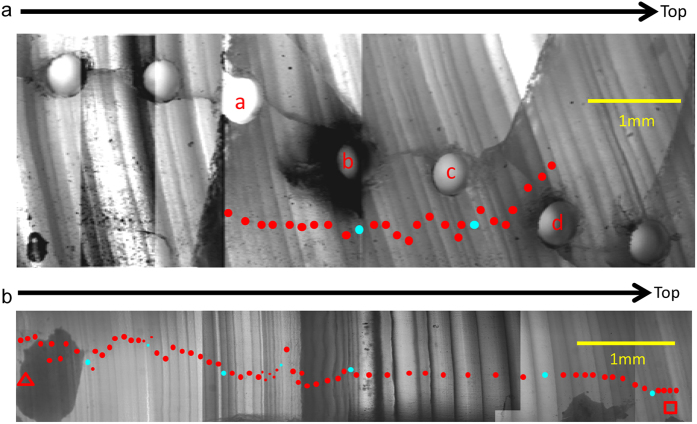
Transmitted light micrographs of thin section of stalagmite XL-1 at the transitions into GIS 14 (**a**) and GIS 15.2 (**b**). The letters a to d are subsample positions for δ^18^O analyses shown in Fig. 2. The triangle and rectangle denote the position of start and end of transition into GIS 15.2, respectively, which are the same as in Fig. 2. Solid red and cyan dots denote annual lamina and every tenth lamina, respectively. There are 27 and 74 annual lamina covering the full δ^18^O shift into GIS 14 and 15.2, respectively.

The transitions into GIS 14 and 15.2 in our record, constrained by lamina counting, lasted 27 (Fig. 4a) and 74 yr (Fig. 4b), respectively, both of which are in excellent agreement with the NGRIP record (20 ± 1 and 100 ± 6 yr, respectively)^12^,^13^. The duration of these abrupt transitions is also roughly consistent with estimates from other stalagmite records from southern China^10^ and Europe^9^,^11^, constrained by ^230^Th dating only. The transition into GIS 14 in the Hulu record, however, appears long and slow^6^, possibly due to low resolution or small hiatus during this period. This indicates that abrupt climate oscillations in China occurred at a similar pace to Greenland and Europe.

Our record is also characterized by weak centennial-scale EASM excursions within GIS 14. Shifts of up to 1.5% at the beginning and middle parts of GIS 14 in our record also present in Greenland ice cores^1^,^2^,^4^,^5^,^6^,^7^, Kleegruben^9^ and Hölloch^11^ stalagmite records. However, as for the Wulu record in southern China, the first shift is not clear, but the second one is well expressed^10^ (Fig. 3). This suggests that the MIS 3 centennial-scale climatic oscillations observed in the Greenland ice cores are also faithfully recorded in some Chinese stalagmites, especially from sites in northern China.

## Discussion

Changes in the Atlantic Meridional Overturning Circulation (AMOC) have been proposed as the direct cause of millennial-scale temperature changes in central Greenland during the glacial period^24^,^25^. It is argued that an injection of freshwater would dramatically reduce the Atlantic thermohaline circulation (THC) and the transport of heat from the tropics to the high latitudes, causing cold events in Greenland. In turn, a reduction in THC likely increases North Atlantic sea ice cover^26^, which would result in a southward shift of the Intertropical Convergence Zone over the Atlantic and the Pacific^27^, and a weakening of the East Asian summer monsoon through air-sea interactions^28^. The close coupling of abrupt climatic oscillations on millennial to decadal timescales in Greenland, Europe and China suggests a rapid climate transmission via the atmosphere rather than by the ocean. As the volatility of wind field is great, it can transmit climatic signals rapidly and then induce rapid global-scale teleconnections^29^,^30^,^31^,^32^. Thus, atmospheric feedbacks would likely spread the anomaly from a sudden change in the THC more quickly and efficiently than ocean processes^29^,^30^,^31^,^32^. As previously suggested, the northern westerlies may be the links between the Atlantic Ocean and the EASM which can swiftly transmit climatic signals to the EASM^33^,^34^,^35^,^36^.

The robust relationship between the Xinglong stalagmite and Greenland ice-core records indicates that Greenland temperature and EASM variability were coupled on millennial to decal timescales during DO 15 and 14. The rapid transmission of abrupt climate signals from the North Atlantic to the EASM supports an atmospheric teleconnection between the regions, most likely via the westerlies.

## Methods

### ^230^Th dating

Subsamples were drilled along the stalagmite growth axis and analyzed on a multi-collector inductively coupled plasma mass spectrometer (Thermo Fisher NEPTUNE PLUS). The procedures are similar to those described in ref. 37. The chemical procedures used to separate uranium and thorium followed those described in ref. 38.

### Lamina-counting age

The lamina between each two neighboring position of ^230^Th date was counted through the whole thin section of the stalagmite under the microscope with transmitted light. The youngest date of lamina-counting age was from that of ^230^Th age.

### δ^18^O analyses

Oxygen isotopic values were measured on a Finnigan MAT-253 mass spectrometer equipped with a Kiel Carbonate Device IV. Inter-laboratory standard TTB1 was run every 10–15 samples. All oxygen isotopic values are reported relative to the Vienna Pee Dee Belemnite (VPDB) standard. Precision of the δ^18^O analyses was better than 0.15% (2σ).

Both the ^230^Th dating and oxygen isotopic measurements were performed in the isotope laboratory of Xi’anjiaotong University.

## Additional Information

**How to cite this article**: Duan, W. *et al*. Onset and duration of transitions into Greenland Interstadials 15.2 and 14 in northern China constrained by an annually laminated stalagmite. *Sci. Rep.* **6**, 20844; doi: 10.1038/srep20844 (2016).

## Supplementary Material

Supplementary Information
