# Supplementary material for: Onset and duration of transitions into Greenland Interstadials 15.2 and 14 in northern China constrained by an annually laminated stalagmite
Source: Sci Rep. 2016 Feb 10;6:20844. doi: 10.1038/srep20844 (PMC4748405; doi:10.1038/srep20844)
Supplement: Supplementary Information [file srep20844-s1.doc]

**Onset and duration of transitions into Greenland Interstadials 15.2 and 14 in northern China constrained by an annually laminated stalagmite**

Wuhui Duan^1★^, Hai Cheng^2^, ^3^, Ming Tan^1^ and R. Lawrence Edwards^3^

^1^Key laboratory of Cenozoic Geology and Environment, Institute of Geology and Geophysics, Chinese Academy of Sciences, Beijing, 100029, China. *E-mail:duanwuhui@mail.iggcas.ac.cn*

^2^Institute of Global Environmental Change, Xi’an Jiaotong University, Xi’an, 710049, China.

^3^Department of Earth Sciences, University of Minnesota, Minneapolis, Minnesota, 55455, USA

**Supplementary Figures**

Figure S1. Location of Xinglong Cave. It is located in Xionglong County, Hebei Province, about 200 km northeast of Beijing city. The figure was drawn using the software CorelDRAW 12.


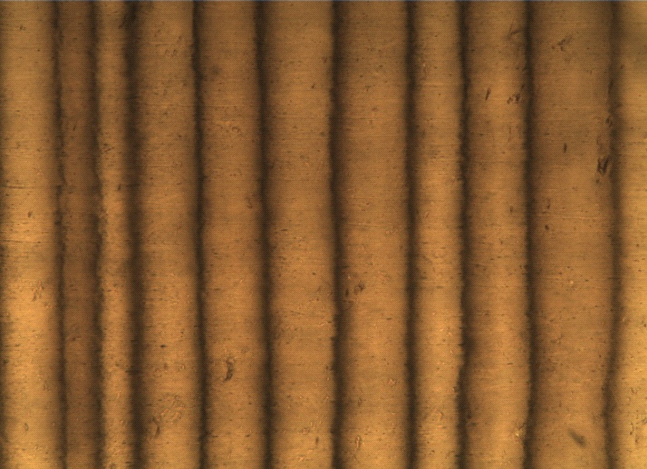


100

μm


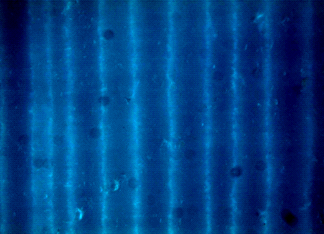


100

μm


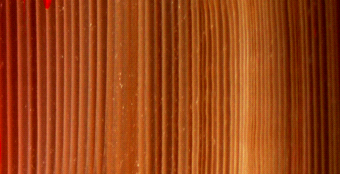


1 mm

b

10 cm

c

d

b

a


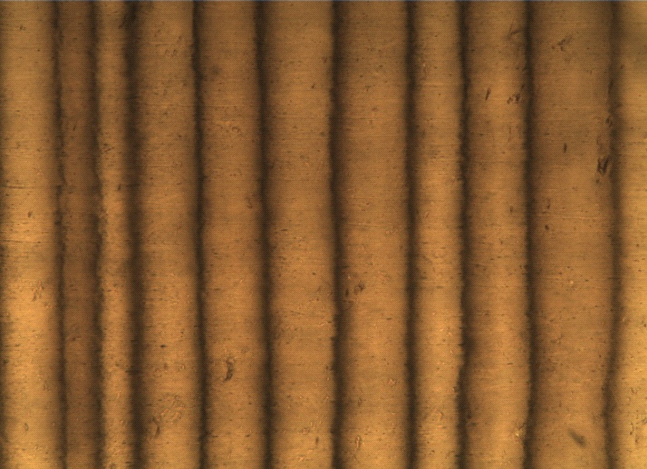


100

μm


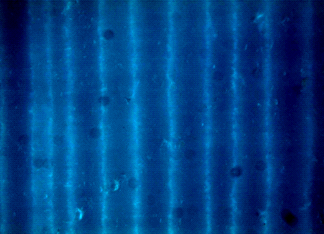


100

μm


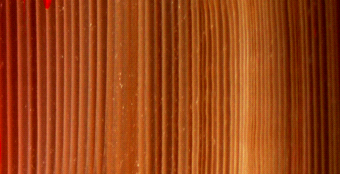


1 mm

b

10 cm

c

d

b

a

Figure S2. The pictures of stalagmite XL-1. a. Thin section of this sample. b, c and d show the micrographs of the laminated structure in reflected, transmitted and fluorescent light, respectively.

The reflected light image shows clear annual lamina of alternating transparent-brown and milky-white sub-layers throughout its length. Under epifluorescence, the milky-white sub-layer shows bright fluorescence. The structure of the lamina in this stalagmite is similar to that from Shihua Cave in Beijing (refs. 1, 2), about 220 km from Xinglong Cave.

Figure S3. Age-depth plot for stalagmite XL-1. The ^230^Th age model was established using the StalAge algorithm (ref. 23 in main text). The green line indicates the derived age models and the red lines represent the 95% uncertainty envelopes. Error bars indicate 2σ uncertainties.

Figure S4. Results of the ‘Hendy test’ (ref. 20 in main text). (a) δ^18^O variations along four growth layers. (b) δ^18^O-δ^13^C correlations along four layers. Different layers are identified by circles (7 mm from the top), diamonds (164 mm), triangles (229 mm) and stars (314 mm), respectively. Along each layer, δ^18^O values are essentially the same, and δ^18^O and δ^13^C values show no statistically significant correlations, suggesting that the stalagmite most likely grew close to isotopic equilibrium (ref. 20 in main text).

| **Supplementary Tables**  Table S1. ^230^Th dating results for stalagmite XL-1 from Xinglong Cave, northern China. Errors are 2σ analytical errors. | | | | | | | | | | | | | | | | | |
| --- | --- | --- | --- | --- | --- | --- | --- | --- | --- | --- | --- | --- | --- | --- | --- | --- | --- |
| **Sample** | **depth** | **^238^U** | | **^232^Th** | | **^230^Th / ^232^Th** | | **δ^234^U^a^** | | **^230^Th / ^238^U** | | **^230^Th Age (yr BP)** | | **^230^Th Age (yr BP)^c^** | | **δ^234^U_Initial_^b^** | |
| **Number** | **(mm)** | **(ppb)** | | **(ppt)** | | **(atomic x10^-6^)** | | **(measured)** | | **(activity)** | | **(uncorrected)** | | **(corrected)** | | **(corrected)** | |
| XL-1-1 | 8 | 122 | ±0 | 609 | ±12 | 3398.9 | ±69.5 | 1656.5 | ±2.7 | 1.0255 | ±0.0027 | 50349 | ±175 | **50299** | **±178** | 1909 | ±3 |
| XL-1-2 | 46 | 129 | ±0 | 772 | ±16 | 2787.8 | ±56.3 | 1578.2 | ±1.7 | 1.0158 | ±0.0018 | 51660 | ±120 | **51598** | **±128** | 1826 | ±2 |
| XL-1-3 | 64 | 107 | ±0 | 1058 | ±21 | 1662.2 | ±33.7 | 1526.4 | ±2.5 | 1.0013 | ±0.0027 | 52073 | ±184 | **51968** | **±198** | 1768 | ±3 |
| XL-1-4 | 78 | 127.7 | ±0.2 | 839 | ±17 | 2505 | ±51 | 1511.3 | ±2.7 | 0.9987 | ±0.0032 | 52299 | ±220 | **52230** | **±225** | 1752 | ±3 |
| XL-1-5 | 99 | 123.7 | ±0.2 | 664 | ±13 | 3006 | ±61 | 1455.2 | ±2.8 | 0.9787 | ±0.0026 | 52491 | ±188 | **52433** | **±193** | 1688 | ±3 |
| XL-1-6 | 115 | 121 | ±0 | 606 | ±12 | 3188.3 | ±64.9 | 1435.8 | ±2.3 | 0.9722 | ±0.0027 | 52583 | ±188 | **52528** | **±192** | 1666 | ±3 |
| XL-1-7 | 124 | 156 | ±0 | 661 | ±13 | 3720 | ±75 | 1393.3 | ±2.3 | 0.9547 | ±0.0020 | 52581 | ±151 | **52531** | **±154** | 1616 | ±3 |
| XL-1-8 | 142 | 171.7 | ±0.2 | 787 | ±16 | 3423 | ±69 | 1379.7 | ±2.4 | 0.9522 | ±0.0023 | 52786 | ±168 | **52735** | **±172** | 1601 | ±3 |
| XL-1-9 | 156 | 148 | ±0 | 708 | ±15 | 3236 | ±67 | 1345.8 | ±2.1 | 0.9396 | ±0.0020 | 52883 | ±152 | **52826** | **±157** | 1562 | ±3 |
| XL-1-10 | 181 | 145.4 | ±0.2 | 630 | ±13 | 3539 | ±74 | 1307.1 | ±2.6 | 0.9304 | ±0.0028 | 53360 | ±209 | **53310** | **±212** | 1520 | ±3 |
| XL-1-11 | 203 | 182 | ±0 | 487 | ±10 | 5795.5 | ±117.5 | 1327.9 | ±2.0 | 0.9412 | ±0.0016 | 53512 | ±126 | **53482** | **±128** | 1545 | ±2 |
| XL-1-12 | 214 | 247.7 | ±0.2 | 582 | ±12 | 6614.7 | ±133.5 | 1314.1 | ±2.0 | 0.9421 | ±0.0014 | 53979 | ±116 | **53952** | **±118** | 1530 | ±2 |
| XL-1-13 | 223 | 282.5 | ±0.5 | 547 | ±11 | 8100 | ±167 | 1325.7 | ±2.7 | 0.9506 | ±0.0038 | 54236 | ±280 | **54214** | **±280** | 1545 | ±3 |
| XL-1-14 | 228 | 315.4 | ±0.3 | 374 | ±8 | 13487 | ±274 | 1367.5 | ±1.9 | 0.9692 | ±0.0016 | 54304 | ±122 | **54290** | **±123** | 1594 | ±2 |
| XL-1-15 | 236 | 278.7 | ±0.3 | 779 | ±16 | 5694 | ±115 | 1353.6 | ±2.0 | 0.9657 | ±0.0015 | 54470 | ±118 | **54439** | **±120** | 1579 | ±2 |
| XL-1-16 | 252 | 225 | ±0 | 1086 | ±22 | 3303.8 | ±66.7 | 1331.6 | ±2.6 | 0.9668 | ±0.0022 | 55203 | ±176 | **55148** | **±180** | 1556 | ±3 |
| XL-1-17 | 258 | 250.6 | ±0.3 | 361 | ±7 | 10825 | ±221 | 1273.8 | ±1.9 | 0.9455 | ±0.0015 | 55452 | ±126 | **55435** | **±126** | 1490 | ±2 |
| XL-1-18 | 271 | 202.7 | ±0.2 | 1239 | ±25 | 2478 | ±50 | 1203.0 | ±1.7 | 0.9187 | ±0.0015 | 55723 | ±125 | **55649** | **±135** | 1408 | ±2 |
| XL-1-19 | 293 | 274 | ±0 | 329 | ±7 | 12877 | ±263 | 1240.4 | ±2.0 | 0.9377 | ±0.0019 | 55940 | ±156 | **55924** | **±156** | 1453 | ±2 |
| XL-1-20 | 311 | 239.3 | ±0.3 | 863 | ±17 | 4120 | ±83 | 1140.4 | ±1.8 | 0.9009 | ±0.0014 | 56439 | ±127 | **56394** | **±130** | 1337 | ±2 |
| XL-1-21 | 334 | 234.3 | ±0.2 | 788 | ±16 | 4327 | ±87 | 1090.0 | ±1.8 | 0.8827 | ±0.0013 | 56737 | ±123 | **56694** | **±127** | 1279 | ±2 |
| XL-1-22 | 370 | 294.9 | ±0.3 | 534 | ±11 | 8032 | ±163 | 1086.4 | ±1.8 | 0.8815 | ±0.0014 | 56771 | ±131 | **56748** | **±132** | 1275 | ±2 |
| ^a^δ^234^U = ([^234^U/^238^U]_activity_ – 1)x1000. ^b^δ^234^U_initial_ was calculated based on ^230^Th age (T), i.e., δ^234^U_initial_ = δ^234^U_measured_ x e^λ234xT^. | | | | | | | | | | | | | | |  |  |  |
| Corrected ^230^Th ages assume the initial ^230^Th/^232^Th atomic ratio of 4.4 ±2.2 x10^-6^. Those are the values for a material at secular | | | | | | | | | | | | | | |  |  |  |
| equilibrium, with the bulk earth ^232^Th/^238^U value of 3.8. The errors are arbitrarily assumed to be 50%. | | | | | | | | | | | | |  |  |  |  |  |
| ^c^B.P. stands for ‘Before Present’ where the “Present” is defined as the year 1950 A.D. | | | | | | | | | | |  |  |  |  |  |  |  |

Table S2. δ^18^O data for stalagmite XL-1 based on the age model of the StalAge algorithm (ref. 23 in main text). Depth is relative to the top (youngest surface) of stalagmite and was measured along the growth axis. b2k: before AD 2000.

| Depth | Date | δ^18^O | Depth | Date | δ^18^O | Depth | Date | δ^18^O |
| --- | --- | --- | --- | --- | --- | --- | --- | --- |
| (mm) | (b2k) | (‰, VPDB) | (mm) | (b2k) | (‰, VPDB) | (mm) | (b2k) | (‰, VPDB) |
| 1 | 50187 | -9.10 | 40 | 51635 | -9.66 | 79 | 52215 | -9.35 |
| 2 | 50218 | -9.39 | 41 | 51649 | -9.97 | 80 | 52226 | -9.61 |
| 3 | 50248 | -9.00 | 42 | 51662 | -9.27 | 83 | 52259 | -9.83 |
| 4 | 50279 | -9.45 | 43 | 51675 | -9.36 | 84 | 52270 | -9.80 |
| 5 | 50311 | -9.74 | 44 | 51689 | -8.88 | 85 | 52280 | -9.29 |
| 6 | 50341 | -9.08 | 45 | 51702 | -8.78 | 86 | 52290 | -9.38 |
| 7 | 50372 | -9.49 | 46 | 51715 | -9.10 | 87 | 52300 | -9.21 |
| 8 | 50402 | -9.64 | 47 | 51728 | -9.28 | 88 | 52310 | -8.89 |
| 9 | 50432 | -8.95 | 48 | 51742 | -9.32 | 89 | 52320 | -9.74 |
| 10 | 50463 | -9.66 | 49 | 51757 | -9.59 | 90 | 52331 | -10.10 |
| 11 | 50495 | -9.07 | 50 | 51773 | -9.73 | 91 | 52342 | -10.01 |
| 12 | 50528 | -9.12 | 51 | 51790 | -9.89 | 92 | 52352 | -10.00 |
| 13 | 50560 | -9.55 | 52 | 51809 | -9.71 | 93 | 52362 | -9.75 |
| 14 | 50592 | -9.62 | 53 | 51829 | -9.60 | 94 | 52372 | -9.91 |
| 15 | 50630 | -9.43 | 54 | 51849 | -9.52 | 95 | 52382 | -9.81 |
| 16 | 50678 | -9.58 | 55 | 51869 | -9.32 | 96 | 52392 | -9.84 |
| 17 | 50715 | -9.27 | 56 | 51889 | -9.29 | 97 | 52401 | -10.09 |
| 18 | 50734 | -9.34 | 57 | 51908 | -9.61 | 98 | 52411 | -9.74 |
| 19 | 50806 | -9.53 | 58 | 51926 | -9.43 | 99 | 52420 | -9.54 |
| 20 | 50984 | -9.41 | 59 | 51943 | -9.61 | 100 | 52430 | -9.34 |
| 21 | 51168 | -9.78 | 60 | 51959 | -9.33 | 101 | 52439 | -10.08 |
| 22 | 51256 | -9.36 | 61 | 51975 | -9.81 | 102 | 52448 | -9.54 |
| 23 | 51291 | -9.88 | 62 | 51990 | -9.85 | 103 | 52457 | -9.78 |
| 24 | 51334 | -9.44 | 63 | 52005 | -9.97 | 104 | 52466 | -9.65 |
| 25 | 51380 | -9.59 | 64 | 52018 | -9.53 | 105 | 52474 | -10.04 |
| 26 | 51414 | -9.41 | 65 | 52031 | -10.06 | 106 | 52483 | -9.50 |
| 27 | 51437 | -9.50 | 66 | 52045 | -9.79 | 107 | 52491 | -9.96 |
| 28 | 51457 | -9.50 | 67 | 52058 | -10.05 | 108 | 52499 | -9.64 |
| 29 | 51474 | -9.57 | 68 | 52071 | -9.70 | 109 | 52507 | -9.86 |
| 30 | 51490 | -9.79 | 69 | 52086 | -9.86 | 110 | 52515 | -10.17 |
| 31 | 51506 | -9.65 | 70 | 52103 | -9.47 | 111 | 52523 | -10.43 |
| 32 | 51523 | -9.43 | 71 | 52119 | -9.99 | 112 | 52532 | -9.99 |
| 33 | 51538 | -10.10 | 72 | 52135 | -9.87 | 113 | 52540 | -9.56 |
| 34 | 51553 | -10.13 | 73 | 52148 | -9.23 | 114 | 52549 | -9.37 |
| 35 | 51567 | -9.96 | 74 | 52160 | -9.49 | 115 | 52558 | -9.40 |
| 36 | 51581 | -9.63 | 75 | 52171 | -9.94 | 116 | 52567 | -9.61 |
| 37 | 51595 | -9.58 | 76 | 52182 | -9.38 | 117 | 52576 | -9.10 |
| 38 | 51608 | -9.54 | 77 | 52193 | -9.06 | 118 | 52584 | -9.42 |
| 39 | 51621 | -9.37 | 78 | 52204 | -8.92 | 119 | 52592 | -9.99 |

Table S2 (*continued*)

| Depth | Date | δ^18^O | Depth | Date | δ^18^O | Depth | Date | δ^18^O |
| --- | --- | --- | --- | --- | --- | --- | --- | --- |
| (mm) | (b2k) | (‰, VPDB) | (mm) | (b2k) | (‰, VPDB) | (mm) | (b2k) | (‰, VPDB) |
| 120 | 52600 | -10.34 | 161 | 53013 | -10.00 | 200 | 53485 | -9.91 |
| 121 | 52607 | -9.71 | 162 | 53027 | -10.22 | 201 | 53505 | -9.71 |
| 122 | 52615 | -9.58 | 163 | 53042 | -10.24 | 202 | 53525 | -10.03 |
| 123 | 52624 | -9.32 | 164 | 53057 | -10.05 | 203 | 53546 | -9.85 |
| 124 | 52632 | -9.29 | 165 | 53072 | -10.07 | 204 | 53569 | -9.82 |
| 125 | 52641 | -9.58 | 166 | 53086 | -9.95 | 205 | 53594 | -9.92 |
| 126 | 52650 | -9.64 | 167 | 53099 | -9.85 | 206 | 53621 | -9.83 |
| 127 | 52659 | -9.62 | 168 | 53111 | -9.87 | 207 | 53654 | -9.51 |
| 128 | 52668 | -9.61 | 169 | 53123 | -9.86 | 208 | 53694 | -9.16 |
| 129 | 52677 | -9.70 | 170 | 53134 | -9.87 | 208.5 | 53716 | -9.04 |
| 130 | 52687 | -9.55 | 171 | 53145 | -10.03 | 209 | 53739 | -8.40 |
| 131 | 52696 | -9.78 | 172 | 53156 | -9.85 | 209.5 | 53761 | -8.90 |
| 132 | 52705 | -9.64 | 172.5 | 53162 | -9.85 | 210 | 53784 | -9.38 |
| 133 | 52714 | -9.44 | 173 | 53168 | -9.13 | 211 | 53829 | -10.15 |
| 134 | 52723 | -9.46 | 173.5 | 53173 | -9.50 | 212 | 53874 | -9.89 |
| 135 | 52732 | -9.53 | 174 | 53179 | -9.99 | 213 | 53917 | -9.32 |
| 136 | 52741 | -9.44 | 175 | 53191 | -9.92 | 214 | 53957 | -9.18 |
| 137 | 52750 | -9.53 | 176 | 53202 | -9.81 | 215 | 53993 | -9.59 |
| 138 | 52760 | -9.87 | 177 | 53214 | -9.93 | 216 | 54025 | -9.67 |
| 139 | 52770 | -10.21 | 178 | 53225 | -9.78 | 217 | 54054 | -9.83 |
| 140 | 52780 | -10.48 | 179 | 53237 | -9.90 | 218 | 54082 | -9.93 |
| 141 | 52790 | -10.20 | 180 | 53248 | -9.48 | 219 | 54109 | -9.18 |
| 142 | 52799 | -9.90 | 181 | 53260 | -10.00 | 220 | 54135 | -9.41 |
| 143 | 52809 | -9.99 | 182 | 53272 | -10.09 | 221 | 54162 | -9.89 |
| 144 | 52820 | -9.71 | 183 | 53283 | -10.28 | 222 | 54190 | -10.05 |
| 145 | 52830 | -9.42 | 184 | 53294 | -10.27 | 223 | 54217 | -9.80 |
| 146 | 52840 | -9.50 | 185 | 53305 | -10.21 | 224 | 54242 | -9.93 |
| 147 | 52850 | -9.55 | 186 | 53317 | -10.06 | 225 | 54263 | -9.63 |
| 148 | 52861 | -9.47 | 187 | 53328 | -9.73 | 226 | 54281 | -9.47 |
| 149 | 52871 | -9.61 | 188 | 53339 | -9.37 | 227 | 54300 | -9.09 |
| 150 | 52883 | -9.54 | 189 | 53349 | -9.73 | 228 | 54325 | -8.55 |
| 151 | 52894 | -9.46 | 190 | 53358 | -10.01 | 229 | 54352 | -8.32 |
| 152 | 52905 | -9.27 | 191 | 53366 | -9.83 | 230 | 54378 | -8.39 |
| 153 | 52916 | -9.64 | 192 | 53375 | -9.41 | 231 | 54403 | -8.49 |
| 154 | 52928 | -10.17 | 193 | 53384 | -9.48 | 232 | 54429 | -8.51 |
| 155 | 52940 | -10.02 | 194 | 53393 | -9.72 | 233 | 54456 | -8.37 |
| 156 | 52952 | -9.70 | 195 | 53404 | -9.89 | 234 | 54487 | -8.63 |
| 157 | 52963 | -9.66 | 196 | 53417 | -10.16 | 235 | 54519 | -8.56 |
| 158 | 52974 | -9.73 | 197 | 53432 | -9.85 | 236 | 54552 | -9.00 |
| 159 | 52986 | -9.72 | 198 | 53448 | -9.77 | 237 | 54587 | -8.86 |
| 160 | 52999 | -9.77 | 199 | 53465 | -9.38 | 238 | 54624 | -8.62 |

Table S2 (*continued*)

| Depth | Date | δ^18^O | Depth | Date | δ^18^O | Depth | Date | δ^18^O |
| --- | --- | --- | --- | --- | --- | --- | --- | --- |
| (mm) | (b2k) | (‰, VPDB) | (mm) | (b2k) | (‰, VPDB) | (mm) | (b2k) | (‰, VPDB) |
| 239 | 54666 | -8.51 | 264 | 55549 | -8.75 | 305 | 56274 | -9.02 |
| 240 | 54718 | -8.61 | 265 | 55568 | -9.09 | 306 | 56293 | -9.44 |
| 241 | 54773 | -8.49 | 266 | 55586 | -9.08 | 307 | 56312 | -9.13 |
| 242 | 54825 | -8.36 | 267 | 55604 | -9.25 | 308 | 56331 | -9.22 |
| 243 | 54875 | -8.42 | 268 | 55621 | -9.20 | 309 | 56350 | -9.46 |
| 244 | 54925 | -8.14 | 269 | 55639 | -9.45 | 310 | 56368 | -9.35 |
| 244.5 | 54951 | -8.86 | 270 | 55656 | -9.55 | 311 | 56386 | -9.26 |
| 245 | 54978 | -8.66 | 271 | 55674 | -9.71 | 312 | 56403 | -9.32 |
| 245.5 | 55006 | -8.68 | 272 | 55691 | -9.65 | 313 | 56420 | -9.21 |
| 246 | 55034 | -8.45 | 273 | 55708 | -9.83 | 314 | 56438 | -9.26 |
| 246.5 | 55061 | -8.58 | 274 | 55725 | -9.18 | 315 | 56455 | -9.17 |
| 247 | 55086 | -8.77 | 275 | 55742 | -9.48 | 316 | 56472 | -9.67 |
| 247.5 | 55108 | -8.84 | 276 | 55759 | -9.69 | 317 | 56488 | -9.98 |
| 248 | 55127 | -9.10 | 277 | 55776 | -9.82 | 318 | 56506 | -9.66 |
| 248.5 | 55145 | -9.12 | 278 | 55792 | -9.76 | 319 | 56522 | -9.56 |
| 249 | 55162 | -9.04 | 279 | 55809 | -9.68 | 320 | 56536 | -9.61 |
| 249.5 | 55179 | -8.80 | 280 | 55826 | -9.78 | 321 | 56550 | -9.76 |
| 250 | 55196 | -8.52 | 281 | 55843 | -9.47 | 322 | 56565 | -10.21 |
| 250.5 | 55213 | -8.52 | 282 | 55860 | -9.80 | 323 | 56579 | -10.26 |
| 251 | 55230 | -8.52 | 283 | 55877 | -10.12 | 324 | 56593 | -9.87 |
| 251.5 | 55246 | -8.67 | 284 | 55894 | -9.93 | 325 | 56608 | -9.85 |
| 252 | 55263 | -8.87 | 285 | 55911 | -9.72 | 326 | 56624 | -10.25 |
| 252.5 | 55280 | -8.56 | 286 | 55929 | -9.96 | 327 | 56640 | -9.60 |
| 253 | 55297 | -8.19 | 287 | 55946 | -9.66 | 328 | 56655 | -10.16 |
| 253.5 | 55315 | -8.11 | 288 | 55964 | -9.79 | 329 | 56671 | -10.21 |
| 254 | 55333 | -8.51 | 289 | 55982 | -10.07 | 330 | 56687 | -10.13 |
| 254.5 | 55350 | -8.13 | 290 | 55999 | -10.37 | 331 | 56704 | -10.19 |
| 255 | 55367 | -8.86 | 291 | 56017 | -9.86 | 332 | 56721 | -9.86 |
| 255.5 | 55382 | -8.65 | 292 | 56034 | -9.52 | 333 | 56734 | -10.29 |
| 256 | 55397 | -9.14 | 293 | 56052 | -8.99 | 334 | 56742 | -10.14 |
| 256.5 | 55411 | -8.59 | 294 | 56069 | -9.21 | 335 | 56751 | -10.10 |
| 257 | 55424 | -8.73 | 295 | 56086 | -8.78 | 336 | 56759 | -9.99 |
| 257.5 | 55437 | -8.44 | 296 | 56104 | -8.18 | 337 | 56767 | -10.01 |
| 258 | 55450 | -8.49 | 297 | 56122 | -8.70 | 338 | 56776 | -9.77 |
| 258.5 | 55461 | -8.85 | 298 | 56141 | -9.00 | 339 | 56784 | -10.08 |
| 259 | 55472 | -8.82 | 299 | 56160 | -8.89 | 340 | 56791 | -10.01 |
| 259.5 | 55482 | -8.87 | 300 | 56178 | -8.57 | 341 | 56798 | -9.97 |
| 260 | 55491 | -8.83 | 301 | 56197 | -8.59 | 342 | 56803 | -10.65 |
| 261 | 55507 | -8.84 | 302 | 56216 | -9.20 | 343 | 56810 | -10.86 |
| 262 | 55520 | -8.62 | 303 | 56236 | -8.78 | 344 | 56816 | -10.80 |
| 263 | 55533 | -8.54 | 304 | 56255 | -8.97 | 345 | 56817 | -10.83 |

Table S 2 (*continued*)

| Depth | date | δ^18^O |
| --- | --- | --- |
| (mm) | (b2k) | (‰, VPDB) |
| 346 | 56817 | -10.44 |
| 347 | 56817 | -10.38 |
| 348 | 56818 | -9.56 |
| 349 | 56818 | -9.64 |
| 350 | 56820 | -9.27 |
| 351 | 56820 | -9.65 |
| 352 | 56822 | -9.77 |
| 353 | 56824 | -10.10 |
| 354 | 56827 | -9.25 |
| 355 | 56829 | -10.49 |
| 356 | 56832 | -10.04 |
| 357 | 56835 | -10.32 |
| 358 | 56837 | -9.77 |
| 359 | 56840 | -10.25 |
| 360 | 56843 | -10.04 |
| 361 | 56845 | -10.54 |
| 362 | 56848 | -10.18 |
| 363 | 56850 | -10.14 |
| 364 | 56853 | -10.21 |
| 365 | 56856 | -10.30 |
| 366 | 56858 | -10.35 |
| 367 | 56861 | -9.95 |
| 368 | 56864 | -10.10 |
| 369 | 56867 | -9.85 |
| 370 | 56869 | -9.93 |
| 371 | 56872 | -9.80 |
| 372 | 56875 | -9.85 |
| 373 | 56879 | -9.91 |
| 374 | 56882 | -9.92 |
| 375 | 56885 | -9.87 |

**References**

1. Tan, M. *et al*. Cyclic rapid warming on centennial-scale revealed by a 2650-year stalagmite record of warm season temperature. *Geophys. Res. Lett.***30**, 1617, doi:10.1029/2003GL017352 (2003).

2. Cai, B., Zhu, J., Ban, F. & Tan, M. Intra-annual variation of the calcite deposition rate of drip water in Shihua Cave, Beijing, China and its implications for palaeoclimatic reconstructions. *Boreas.***40**, 525-535 (2010).
